# The immunomodulatory effect of microglia on ECM neuroinflammation via the PD‐1/PD‐L1 pathway

**DOI:** 10.1111/cns.13760

**Published:** 2021-11-11

**Authors:** Yan Shen, Yinghui Li, Qinghao Zhu, Jun Wang, Yuxiao Huang, Jiao Liang, Xingan Wu, Ya Zhao

**Affiliations:** ^1^ Department of Medical Microbiology and Parasitology Fourth Military Medical University Xi’an China

**Keywords:** experimental cerebral malaria, microglia, neuroimmune, PD‐1/PD‐L1

## Abstract

**Introduction:**

The experimental cerebral malaria (ECM) model in C57BL/6 mice infected with *Plasmodium berghei* ANKA (PbA) has revealed microglia are involved in the ECM immune microenvironment. However, the regulation of microglia in the ECM immune response is not clear, and there is no safe and efficient treatment clinically for the protection of the nerve cells.

**Aims:**

To elucidate the negative regulation mechanism in the ECM brain mediated by microglia. Furthermore, to investigate protective effect of the appropriate enhancement of the PD‐1/PD‐L1 pathway in the brain against ECM through the intrathecal injection of the adenovirus expressing PDL1‐IgG1Fc fusion protein.

**Results:**

The PD‐1/PD‐L1 pathway was induced in the ECM brain and showed an upregulation in the microglia. Deep single‐cell analysis of immune niches in the ECM brainstem indicated that the microglia showed obvious heterogeneity and activation characteristics. Intrathecal injection of recombinant adenovirus expressing PD‐L1 repressed the neuroinflammation and alleviated ECM symptoms. In addition, the synergistic effect of artemisinin and intracranial immunosuppression mediated by PD‐L1 was more efficacious than either treatment alone.

**Conclusion:**

The appropriate enhancement of the PD‐1/PD‐L1 pathway in the early stage of ECM has an obvious protective effect on the maintenance of immune microenvironment homeostasis in the brain. Regulating microglia and the PD‐1/PD‐L1 pathway could be considered as a promising approach for protection against human cerebral malaria in the future.

## Graphical abstract

Microglia activated and expressed PD‐L1 and PD‐1 induced by PbA infection.The PD‐L1 could interacted with PD‐1 on the infiltrated CTL cells and inhibited the killing activity of CTL cells.The intrathecal injection of expression vector PDL1‐IgGFc could inhibit intracerebral inflammation and improved ECM.

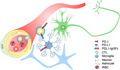

## 1 INTRODUCTION

Cerebral malaria (CM), caused by *Plasmodium falciparum* infection, is one of the most serious forms of malaria. Clinical studies have found that most patients with CM can quickly develop central nervous system (CNS) symptoms, and an excessive inflammatory response is the chief cause of damage to the blood‐brain barrier (BBB) and the occurrence of CM.^1^ The current treatment of CM is limited to artemisinin‐based combination therapy (ACT) and emergency support due to the lack of targeted neuroprotective and vascular therapies. Excessive inflammatory responses in CNS may prevent homeostasis of the immune microenvironment, leading to the failure of antimalarial drug therapy.

Microglia, brain‐resident macrophages,^2^ proliferated in the early stage in the experimental cerebral malaria (ECM) model infected with *Plasmodium berghei* ANKA (PbA), upregulated the inflammatory level,^3^ and aggravated CM nerve inflammation^4^; activated microglia could closely interact with the parasite‐specific T cells around the microvessels^5^ and impacted the physiological migration of nerve cells.^6^ By regulating the immune state of microglia, a balance could be struck between the CM immune defense response and immune‐pathological injury.

A murine cytomegalovirus‐induced encephalitis study found that activated microglia, via the PD‐L1 pathway, inhibit the IFN‐γ and IL‐2 expression in CD8^+^ T cells.^7^ Microglia could overexpress PD‐L1, leading to T cell dysfunction and apoptosis in glioblastoma.^8^ Surgical brain injury research has shown that activation of PD‐1/PD‐L1 signaling with exogenous PD‐L1 protein could significantly reduce the inflammatory response and brain edema.^9^ These results suggest that the PD‐1/PD‐L1 pathway on microglia played an important role in the immune regulatory mechanism of the nervous system.

We hypothesized that microglia may be involved in the regulation of ECM‐immune niches through the PD‐1/PD‐L1 pathway and further enhancement of the PD‐1/PD‐L1 pathway in the brain could reduce inflammatory damage and affect the outcome of ECM. To test this hypothesis, mice were intrathecal injected (i.t) with the PD‐L1 adenovirus expression vector specific and showed a neuroprotective effect against ECM. We proposed that microglia participate in the ECM intracranial inflammatory response and could inhibit the T cells by expressing PD‐L1.

## 2 METHODS

### 2.1 Ethics statement

All animal experiments were approved by the Institutional Review Board of the Fourth Military Medical University (No: IACUC‐20200401). All efforts were made to minimize the suffering of animals employed in this study.

### 2.2 Parasites and infection

*Plasmodium berghei* ANKA was maintained and used as previously reported in our laboratory. All mice were injected intraperitoneally (i.p.) with 5 × 10^6^ parasitized‐infected red blood cells (pRBCs). Parasitemia was determined from Giemsa‐stained thin blood smears. The mice were monitored daily for symptoms of ECM. ECM mice may have symptoms, such as loss of appetite and weight, dull hair, and sluggish movement (Video S1). To evaluate the pathological changes in the brain during the onset of ECM, the brain was divided into four parts: the olfactory bulb, cerebellum, cerebrum, and brainstem. For experiments with multiple groups, all mice were first infected, and then randomly assigned to treatment groups.

### 2.3 Mice and groups

A total of 159 mice were sacrificed for this study, all of which were C57BL/6 male mice (5–6 weeks old and 18–20 g in weight), including 118 mice used for infection experiments^10^ and 41 mice used as normal controls. All mice were bred and housed under specific pathogen‐free conditions.

Different time groups were used, respectively, on the 3, 4, 5, 6, and 7 days after PbA infection. The intrathecal injection was performed in three infected groups, separately injected with empty vector adenovirus, IgG1Fc adenovirus, or PDL1‐IgG1Fc adenovirus. The normal mice were used as blank control, and the infected mice was used as the positive control.

In the experiments combining artemisinin with PD‐L1, infected mice were treated with artemisinin combined with PDL1‐IgG1Fc adenovirus i.t, artemisinin combined with IgG1Fc adenovirus i.t, artemisinin combined with empty vector virus i.t, artemisinin, or PDL1‐IgG1Fc adenovirus i.t, respectively. The normal mice were used as blank control, and untreated infected mice were used as the positive control. The dose of artemisinin was 0.5 mg per mouse at a time and injected intraperitoneally twice on the 4th and 5th days after infection.

### 2.4 Antibodies and reagents

The primary antibodies including anti‐mouse GFP antibody (GB13227, 1:50), anti‐mouse Ki67 antibody (GB111141, 1:1000), anti‐mouse PD‐L1 antibody (GB13339, 1:300), anti‐mouse PD‐1 antibody (GB13338, 1:200), anti‐mouse iNOS antibody (GB11119, 1:500), anti‐mouse IL‐6 antibody (GB11117, 1:500), anti‐mouse CD8α antibody (GB13429, 1:200), and anti‐mouse MCP‐1 antibody (GB11191, 1:500) and the fluorescence‐labeled secondary antibodies, including FITC‐conjugated goat anti‐mouse IgG (H + L) (GB22301, 1:50), FITC‐conjugated goat anti‐rabbit IgG (H + L) (GB22303, 1:50), Cy3‐conjugated goat anti‐mouse IgG (H + L) (GB21301, 1:100), and Cy3‐conjugated goat anti‐rabbit IgG (H + L) (GB21303, 1:100) for immunofluorescence (IF) staining were purchased from Servicebio. APC‐labeled antibodies against mouse PD‐1 (109111, 1:100), FITC‐labeled antibodies against mouse CD11b (101205, 1:100), and PE‐labeled antibodies against mouse CD45 (103105, 1:200) and CD274 (B7‐H1, PD‐L1) (124307, 1:200) were purchased from BioLegend. PD‐1/CD279 (66220‐1‐Ig, 1:1000), PD‐L1/CD274 (66248‐1‐Ig, 1:1000), amyloid‐β (β‐APP) (25524‐1‐AP, 1:1000), and MCP‐1 (66272‐1‐Ig, 1:1000) were measured using western blot, purchased from Proteintech. The TUNEL detection kit (11684817910) (using the concentration with recombinant TdT enzyme/CF488‐dUTP labeling mix/equilibration buffer = 1:5:50) was purchased from Servicebio. All these antibodies and reagents were used with the schedules and doses indicated in the manufacturer's manual.

### 2.5 Quantitative PCR

After anesthesia with 1% pentobarbital sodium i.p. (50 mg/kg), the mice were perfused through the heart with 10‐ml preheated 0.9% NaCl solution to remove blood, and the whole brain was extracted and crushed. Total RNA was extracted using RNAiso Plus (TaKaRa Bio) according to the manufacturer's protocol. The PrimeScript RT Master Mix kit (TaKaRa Bio) was used to prepare cDNA from total RNA following standard protocols. Quantitative PCR was performed on a real‐time PCR system (Vazyme) using synthetic primers (Table S1). Samples were subjected to 40 cycles of amplification at 95°C for 5 s and 60°C for 15 s after an initial hold at 95°C for 30 s. Relative expression data were calculated using the 2^−ΔΔCT^ method. The transcriptional level of β‐action was used as control.

### 2.6 Purification of leukocytes in the brain parenchyma

The mice were euthanized and perfused through the heart with 10‐ml 0.9% NaCl solution to remove non‐adhered RBCs and leukocytes from the cerebrovascular. The brains were removed, cut into small pieces, and crushed into a single cell in an RPMI medium. The brain homogenates were prepared into a 10‐ml RPMI medium with a 30% Percoll gradient medium (GE Healthcare 17‐0891‐02). The pelleted cells were further purified in 2 ml of 70% Percoll gradient medium. The upper Percoll layers, containing myelin debris and cells other than leukocytes, were carefully removed. The cells at the layered interface were carefully collected using a Pasteur pipette and washed in 8‐ml D‐Hanks. After centrifugation, leukocytes were prepared for staining.

### 2.7 Flow cytometry

The cells were resuspended in 200 µl of FACS buffer (D‐Hanks with 0.5% FBS) over a 40‐μm strainer. The antibodies were added at the concentrations described above. Cells were incubated at 4°C for 30 min in the dark, washed twice and resuspended in 400 µl of FACS buffer. The detection was used flow cytometry (BD FACSCanto) based on fluorescent labeling of the antibody, and the data were analyzed using Flow Jo 7.6.1. Each sample collected was 0.1–1 × 10^5^ cells.

### 2.8 BBB integrity assay

In vivo, BBB permeability was checked using an Evans Blue (EB) permeability assay. The mice were injected with 100 µl of 1% EB in PBS i.p. After 8 h, the mice were lethally anesthetized and perfused intracardially with 20‐ml 0.9% NaCl solution to remove intravascular EB. Each brain was removed, weighed, and incubated in 0.5‐ml formamide overnight at 56°C. The amount of extracted EB was determined using photometric analysis of the EB‐formamide solution at 620 nm and by comparison with an EB standard curve.

### 2.9 Histology

To prepare paraffin sections of the brain, the mice were perfused with 10‐ml 0.9% NaCl solution. The brains were carefully extracted and fixed in 4% PFA overnight. Dehydration was performed using sequential alcohol washes with 50%, 70%, 90%, and 100% ethanol. Xylene was used to perforate the brain tissue. The brains were molded using paraffin wax, and 5‐μm tissue sections were prepared, collected on poly‐L‐lysine‐coated slides, and IF stained.

### 2.10 Immunofluorescence staining

After dewaxing and rehydration, paraffin sections were treated with Citrate Antigen Retrieval Solution (BBI E673001), and then blocked with blocking buffer (2% FBS, 3% BSA, and 0.2% Triton X‐100 in PBS). The sections were incubated with primary antibodies diluted in blocking buffer overnight at 4°C (isotype‐matched antibodies were used as controls). After washing thrice with PBST (PBS with 0.2% Triton X‐100), the sections were incubated with secondary antibodies diluted with blocking buffer for 2 h at 25–30°C in the dark. The nuclei were stained with a DAPI staining solution (Servicebio G1012) for 10 min. Slides were mounted with Antifade (Servicebio G1401). The IF‐stained sections were examined using digital slide scanning (Pannoramic DESK, P‐MIDI, P25) and analyzed using CaseViewer 2.4 (3DHISTECH).

### 2.11 Recombinant adenovirus that expresses the PDL1‐IgG1Fc fusion protein

Primers for murine PD‐L1 and IgG1Fc were designed and synthesized based on the published sequence:

**Table:** 

PD‐L1	F: 5´‐CAGAGATCTATGAGGATATTTGCTGGCATT‐3´ R: 5´‐CTCGAATTCGTGAGTCCTGTTCTGTGGAGG‐3´
IgG1Fc	F: 5´‐GACGAATTCGTGCCCAGGGATAGTGGTAGTAAGCCTAGCATAAGTA CAGTCCCAGAAGTATCATCT‐3´ R: 5´‐ATTCCGCGGTCATTTACCAGGAGAGTGGGA‐3´

cDNAs encoding the mouse PD‐L1 extracellular domain and IgG1Fc were generated using RT‐PCR from mouse T cell mRNA. Cysteines in the hinge region were replaced with serines, and the PD‐L1 fusion protein has a low ADCC or antibody‐mediated opsonophagocytosis effect. After digestion with EcoR I and Bgl II and gel purification, the PCR products were fused to the Fc domain of mouse IgG1Fc. The fusion genes were transfected into a recombinant adenovirus. Then, 293 T cells were infected with this recombinant adenovirus, and the supernatant was collected and detected using western blotting with anti‐PD‐L1 mAb to measure PDL1‐IgG1Fc.

### 2.12 Single‐cell RNA‐seq

After the heart perfusion with saline to remove non‐adhered cells, the brainstem of mice was collected and dissociated. The live cells were collected and analyzed using single‐cell RNA‐seq. The cells were loaded onto a 10X Genomics Chromium chip per factory recommendations. Reverse transcription and library preparation were performed using the 10X Genomics Single‐Cell v2 kit following the 10X Genomics protocol. Five high‐quality single‐cell transcriptomes (three in the ECM group and two in the normal group) with t‐distributed stochastic neighbor embedding (*t*‐SNE) projection were obtained.

### 2.13 Intrathecal injection

The back of the mice was shaved after anesthesia with 1% pentobarbital sodium i.p (50 mg/kg). Intrathecal injection of 5‐µl recombinant adenovirus (1 × 10^9^ pfu/ml) was performed in the anterior line of the double sacrum on the midpoint of the skin (spinous process) using a 25‐µl microsyringe. The spot was pressed for 1 min after injection.

### 2.14 Statistical analyses

All the data were processed and analyzed with GraphPad Prism software 9.0. The data were expressed as mean ± standard deviation (SD). One‐way ANOVA was used for multiple‐group comparisons, and an unpaired *t*‐test was used for comparison between two groups. Statistical difference for survival curve was analyzed using the log‐rank test. *p*‐values of <0.05 were considered significant.

## 3 RESULTS

### 3.1 PbA infection induced the PD‐1/PD‐L1 pathway in the brain

**FIGURE 1:**
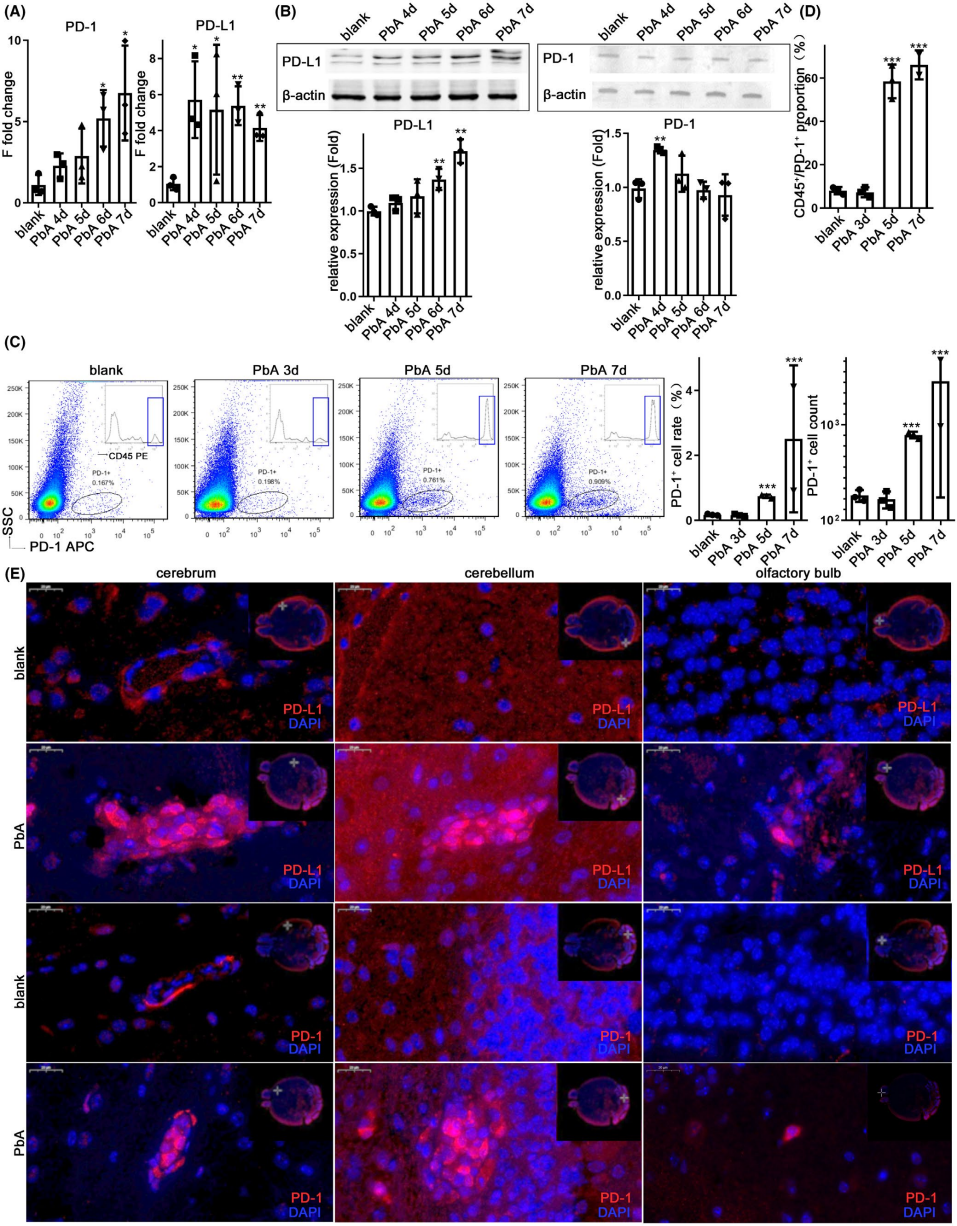
PbA infection induced the PD‐1/PD‐L1 pathway in the brain. The mRNA levels (A) and protein levels (B) of PD‐1 and PD‐L1 in the brain continued to increase 4–7‐day post‐infection (dpi). The microglia were isolated from the infected mice 5–7 dpi or WT mice, followed by anti‐PD‐1 staining. Results of flow cytometry analysis; the percentage and number of PD‐1^+^ cells analyzed using flow cytometry show an increase in PD‐1^+^ cells in ECM mice (C), most of which are CD45^+^(D). (E) Immunofluorescence (IF)‐stained brain sections at 7 dpi show PD‐1^+^ and PD‐L1^+^ cells in the brain of ECM mice. Results in (A–D) are expressed as the mean ± SD of three independent experiments. *p < 0.05, **p < 0.01, and ***p < 0.001 indicate that the differences are significant (unpaired *t*‐test, *n* = 3)

The expression of PD‐1/PD‐L1 in human neuroimmune cells was extremely low or undetectable under physiological conditions.^11^ Using C57BL/6 mice infected with PbA, the transcription and protein level of PD‐1 and PD‐L1 were detected separately using qPCR and western blotting, and first verified to be involved in the ECM brain. The transcriptional level of PD‐1 in the brain continued to increase with the prolongation of infection time compared with that in the normal group, and it showed significant differences on the 6th day (*p* = 0.0184) and 7th day (*p* = 0.0304) of infection. The level of β‐actin was used as control. The level of PD‐L1 increased by sixfold (*p* = 0.0199) on the 4th‐day early stage of infection, while the mice did not show any neurological symptoms, and maintained a high level on the 5th day (*p* = 0.0184), 6th day (*p* = 0.0027), and 7th day (*p* = 0.0025) (Figure 1A). The protein level was upregulated post‐infection, and PD‐L1 showed a significant difference on the 6th day (*p* = 0.0085) and 7th day (*p* = 0.0012) post‐infection, while the PD‐1 level slightly elevated on the 4th day (*p* = 0.0026) (Figure 1B), the protein level of β‐actin as control.

The microglia were separated from whole brain tissue using Percoll density gradient centrifugation,^12^ among which the microglia were the majority in the extracted cells, while other white blood cells were also extracted. The rate of PD‐1^+^ cells among the extracted cells increased visibly on the 5th day (*p* < 0.001) and 7th day (*p* < 0.0001) after infection; the quantity of PD‐1^+^ cells increased on the 5th day (*p *= 0.0001) and 7th day (*p *= 0.0002) after infection, comparing with the normal mice (Figure 1C). Further analysis showed that the proportion of CD45^+^ cells in PD‐1^+^ cells increased significantly on the 5th day (*p* = 0.0004) and 7th day (*p* = 0.0006) after infection (Figure 1C,D). The IF staining of brain tissue sections showed that PD‐1^+^ cells and PD‐L1^+^ cells were widely observed in the cerebrum, cerebellum, and olfactory bulb of ECM mice on the 7th day, and mainly located in the cerebral microvascular obstruction (Figure 1E; Figure S1A).

### 3.2 Microglial activation and upregulation of the PD‐1/PD‐L1 signaling pathway in the ECM brain

**FIGURE 2:**
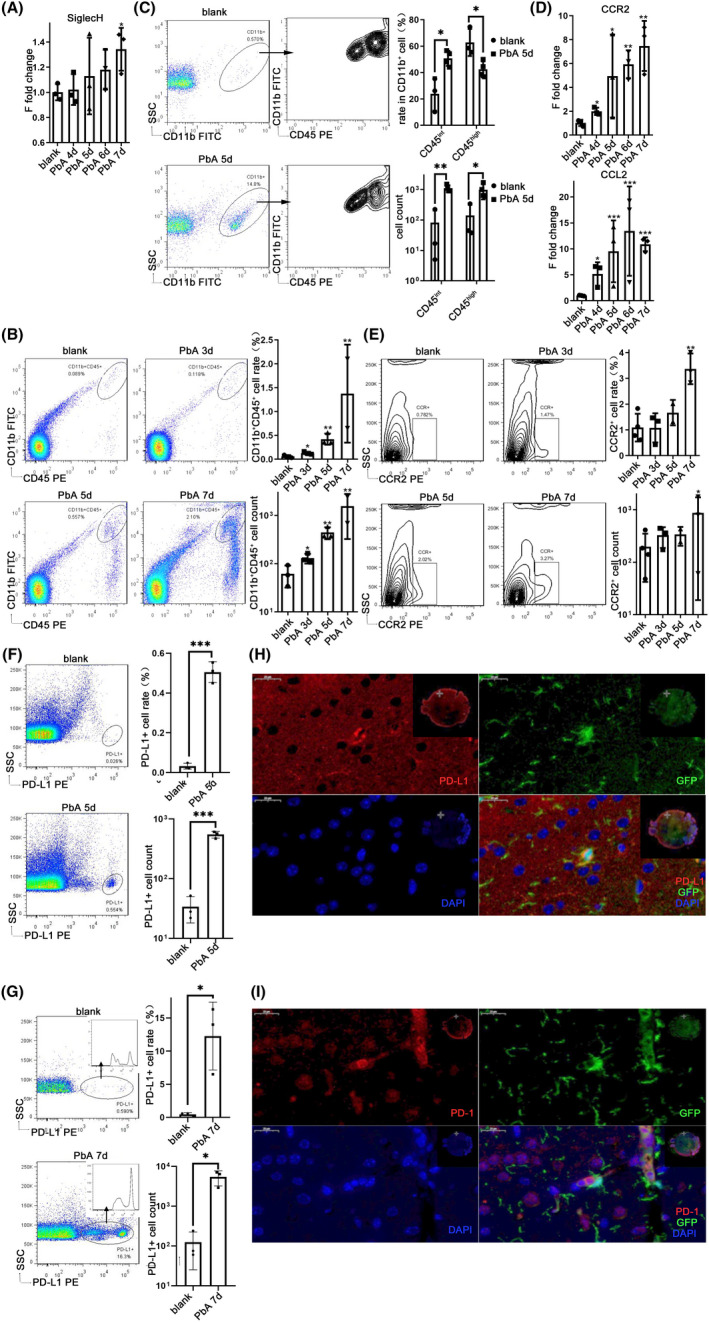
Microglial activation and upregulation of the PD‐1/PD‐L1 signaling pathway in the brain of ECM. The brains were harvested from 4 to 7 dpi. (A) qPCR shows that the expression level of Siglec‐H increased on day 7. The microglia were isolated from the brain and co‐incubated with anti‐CD11b and anti‐CD45 mAbs. Flow cytometry analysis shows that CD11b^+^CD45^+^ cells in the ECM brain increased (B), and CD45^int^ cells among CD11b^+^ cells significantly increased after infection (C). CX3CR1‐GFP mice were infected with PbA and the brains were captured. qPCR shows that the expression levels of macrophage chemokine CCR2 and CCL2 increased (D); the CCR2^+^ cells increased in the ECM brain using flow cytometry analysis (E). Flow cytometry analysis shows that the PD‐L1^+^ cells in the brain increased at 5 dpi (F) and 7 dpi (G) with PbA. IF‐stained CX3CR1‐GFP brain sections at 7 dpi demonstrate that GFP^+^ and PD‐L1^+^ have co‐localization (H) and GFP^+^ cells are close to PD‐1^+^ cells (I). The results in (A–G) are expressed as the mean ± SD of three independent experiments. *p < 0.05, **p < 0.01, and ***p < 0.001 indicate that the differences are significant (unpaired *t*‐test, *n* = 3)

To clarify the obvious activation and expression profile changes in microglia in the ECM brain, qPCR was used to detect the transcriptional levels of microglial markers in the brain. There was a significant upregulation in the transcriptional level of Siglec‐H,^13^ expressed in both mature and activated microglia, on the 7th day after infection (*p* = 0.0317) (Figure 2A). However, TMEM119,^14^ a marker of mature microglia, and the marker of resting microglia P2Y12 receptor^15^ showed no difference (Figure S2A). In addition, the transcriptional levels of MHC‐II IAb increased significantly on the 6th day (*p* = 0.0006) and 7th day (*p* = 0.0014) post‐infection, while MHC‐II IEb also showed a noteworthy increase on the 7th day after infection (*p* = 0.0033), with no obvious changes in the transcription level of MHC‐I molecules (Figure S2B).

The cells extracted from the brain tissue of PbA‐infected mice were confirmed using flow cytometry. Compared with the normal mice, the proportion of CD11b^+^CD45^+^ cells in the ECM brain showed a significant increase on the 3rd day (*p* = 0.0425) post‐infection and increased continuously on the 5th day (*p* = 0.0065) and 7th day (*p* = 0.0015), while the increased quantity of CD11b^+^CD45^+^ cells was obvious on the 3rd day (*p* = 0.0476), 5th day (*p* = 0.0063), and 7th day (*p* = 0.0012) after infection (Figure 2B). Further analysis showed that CD11b^+^CD45^+^ cells were divided into two groups: CD11b^+^CD45^high^ and CD11b^+^CD45^int^. Compared with the normal group, the proportion of CD45^int^ cells in CD11b^+^ cells increased on the 5th day after infection (*p* = 0.0120), while the proportion of CD45^high^ cells in CD11b^+^ cells decreased (*p* = 0.0208). Compared with the normal group, the quantity of CD45^int^CD11b^+^ cells increased (*p* = 0.0021), and the quantity of CD45^high^CD11b^+^ cells also increased (*p* = 0.0434) (Figure 2C).

CX3CR1‐GFP mice specifically expressed GFP in microglia, monocytes, macrophages, and dendritic cells. The CX3CR1‐GFP mice infected with PbA confirmed that the microglia were significantly activated, and their expression profile changed.^3^ The transcription levels of microglia‐related chemokine CCR2 in the ECM brain increased on the 4th day (*p* = 0.0144) and continued to be upregulated on the 5th day (*p* = 0.0111), 6th day (*p* = 0.0021) and 7th day (*p* = 0.0061) after PbA infection, using qPCR. The transcriptional level of macrophage chemokine CCL2 (MCP‐1) was also significantly upregulated on the 4th day (*p* = 0.0328), 5th day (*p* = 0.0004), 6th day (*p* = 0.0002), and 7th day (*p* = 0.0002) after infection (Figure 2D). The cells extracted from the brain tissue of CX3CR1‐GFP mice were detected using flow cytometry. The proportion of CCR2^+^ cells in the separated cells showed a noteworthy increase on the 7th day in the ECM brain compared with that in the normal group (*p* = 0.0032), and the quantity of CCR2^+^ cells also increased after infection (*p* = 0.0202) (Figure 2E). There was no significant difference in the percentage of CX3CR1‐GFP^+^ cells in the brain with increasing infection time (Figure S2C). Further analysis of these cells revealed that they were also divided into two subsets based on the level of CD45 expression, CD45^high^ and CD45^int^ subgroups. The proportion of CD45^int^ cells in CX3CR1‐GFP^+^ cells tended to decrease with time of infection (*p* = 0.0007), while the proportion of CD45^high^ cells in CX3CR1‐GFP^+^ cells increased (*p* = 0.0023) (Figure S2C).

Flow cytometry analysis also confirmed there were only a small number of PD‐L1^+^ cells under physiological conditions. On the 5th day after infection, a cluster of PD‐L1^+^ cells appeared in the cells extracted from the brain (Figure 2F). The proportion of PD‐L1^+^ cells (*p* = 0.0163) and the quantity of PD‐L1^+^ cells (*p* = 0.0146) increased significantly with the infection of PbA on the 7th day, and the PD‐L1^+^ cells were divided into two groups (Figure 2G). The detection of cells extracted from the brain tissue of CX3CR1‐GFP mice using flow cytometry also found a subgroup of microglia with the characteristic of GFP and CD45 double‐positive, which showed a high expression of PD‐L1 (Figure S2D). IF staining of brain tissue sections confirmed that PD‐L1 was expressed in CX3CR1‐GFP^+^ cells on the 7th day after infection (Figure 2H), and the CX3CR1‐GFP^+^ cells were close to PD‐1^+^ cells (Figure 2I), while few PD‐1^+^ or PD‐L1^+^ cells were found in the normal brain (data not shown). IF staining also indicated that the quantity of Ki67^+^CX3CR1‐GFP^+^ cells increased after PbA infection (*p* = 0.0077) (Figure S2E), while it was very low in the physiological brain (data not shown).

### 3.3 The changes in PD‐1 and PD‐L1 were different in each region of the brain

**FIGURE 3:**
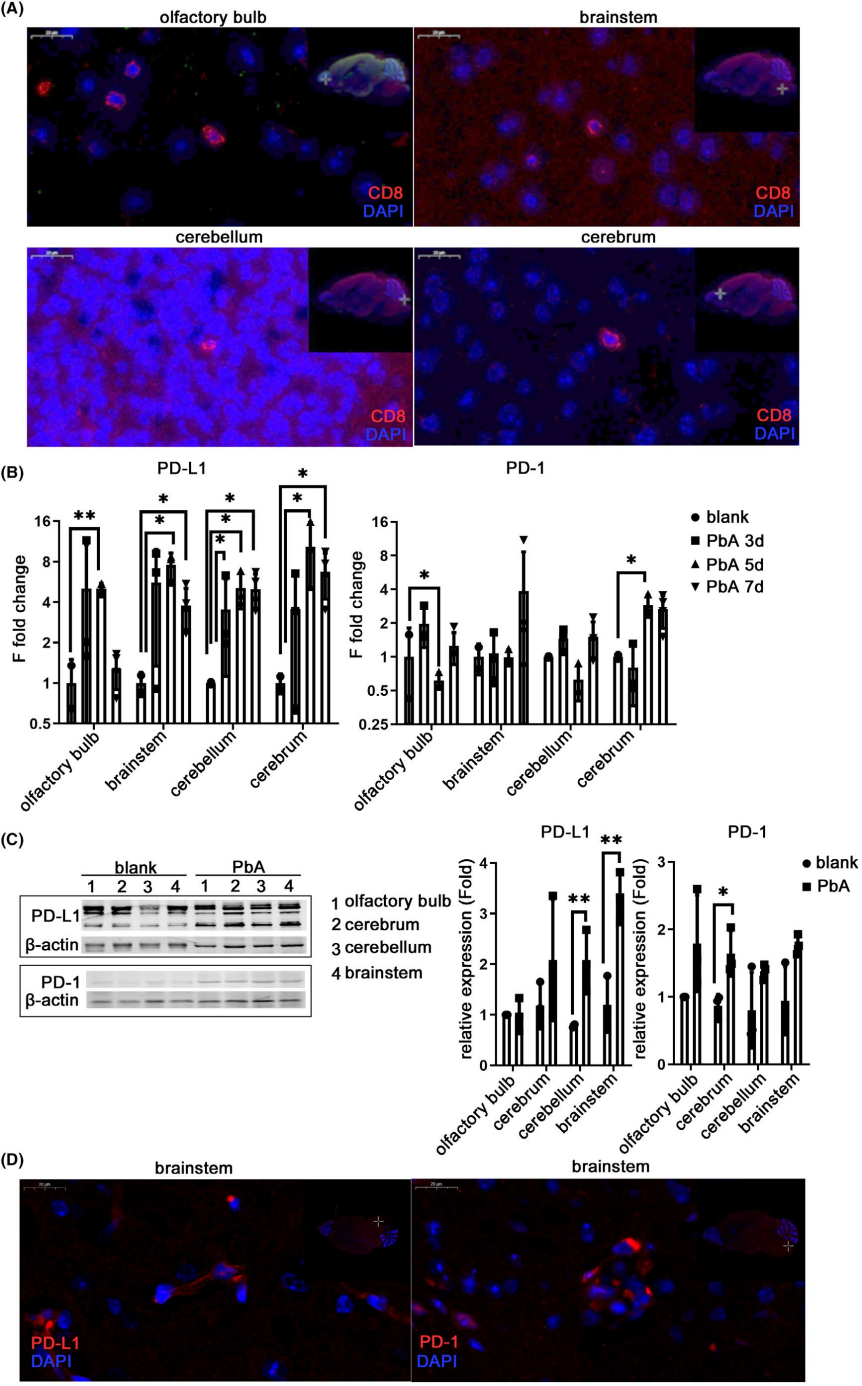
Changes in PD‐1 and PD‐L1 in the brainstem of ECM mice. C57BL/6 mice were infected with PbA, and each region of the brain was harvested from 4 to 7 dpi. IF‐stained brain sections at 7 dpi show infiltrating CD8^+^ cells in each region of the ECM brain (A). The mRNA (B) and protein levels (C) of PD‐L1 and PD‐1 in parts of the brain increased. The IF‐stained brain sections show the expression of PD‐L1 and PD‐1 in the brainstem (D). The results in (B–C) are expressed as the mean ± SD of three independent experiments. *p < 0.05, **p < 0.01, and ***p < 0.001 indicate that the differences are significant (unpaired *t*‐test, *n* = 3)

Patients with CM have diffuse brain lesions, and the neurological manifestations are nonspecific. Most of the patients who died from CM showed acute hemorrhagic infarction, which was found in the brainstem, thalamus, cerebellum, brain, and hippocampus^16^ and is related to obvious infiltration of CD8^+^ T cells. The infiltrating CD8^+^ cells were revealed through IF staining of ECM brain tissue sections on the 7th day after infection, which were very few but were distributed in the olfactory bulb, cerebrum, cerebellum, and brainstem (Figure 3A). Owing to the complex structure and strict functional division of the nervous system, the regulation of the PD‐1/PD‐L1 pathway and expression of its members in different brain regions may be unequable. qPCR was used to detect the transcriptional levels of PD‐1 and PD‐L1 in different brain regions. The transcription level of PD‐L1 increased and reached a peak on the 5th day after infection in the olfactory bulb (*p* = 0.0025), brainstem (*p* = 0.0141), and cerebrum (*p* = 0.0448), and there was a significant increase in the cerebellum from the 3rd day (*p* = 0.0154). The increase in the transcription level of PD‐1 was not as obvious as PD‐L1, and significant upregulation was found only in the olfactory bulb (*p* = 0.0307) and cerebrum (*p* = 0.0287) (Figure 3B). The protein levels of PD‐1 and PD‐L1 in different brain regions were detected by western blotting on the 7th day post‐infection. The protein level of PD‐L1 increased significantly in the brainstem (*p* = 0.0073) and the cerebellum (*p* = 0.0050), while a significant increase in PD‐1 protein was detected only in the cerebrum (*p* = 0.0237) on the 7th day after infection (Figure 3C). The expression of PD‐1 and PD‐L1 in the brainstem was observed through IF staining on the 7th day after infection (Figure 3D). The expression of the inflammatory cytokine IL‐6 and iNOS was observed in the ECM brain through IF staining (Figure S3A,S3B).

### 3.4 Single‐cell analysis of immune niches in the brainstem of ECM mice

**FIGURE 4:**
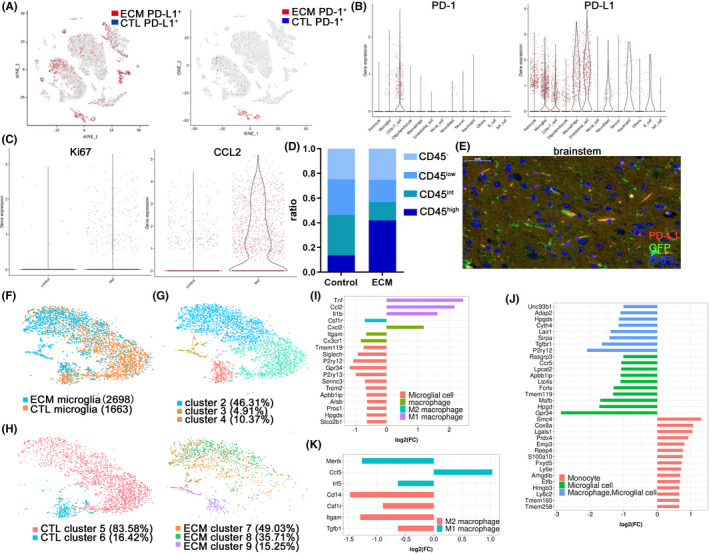
Single‐cell analysis of immune niches in the brainstem of ECM mice. (A) A *t*‐SNE plot of 9607 individual cells of the brainstem in WT mice (*n* = 2) and 6,812 individual cells of the brainstem in ECM mice (*n* = 3) expressed PD‐1 or PD‐L1. Each dot represents a single cell. The color corresponds to the level of transcription. (B) Combined log‐normalized expression values of PD‐L1 or PD‐1 in various cell types of the brainstem in ECM mice based on t‐SNE data. (C) Expression values of Ki67 and CCL2 in microglial cells of the brainstem in ECM mice compared with those in the WT mice. (D) Expression of CD45 in microglia increased significantly in the ECM brainstem and the proportion of CD45^high^ cells increased. (CD45^low^, CD45^int^, and CD45^high^ indicates log2 < 1, 1 ≤ log2 value of <2, and log2 value of ≥2, respectively). (E) IF‐stained CX3CR1‐GFP brain sections at 7 dpi demonstrate that microglia expressed PD‐L1 in the brainstem (asterisk). The microglia showed heterogeneity between the ECM and WT mice (F) and were divided into three clusters (G). (H) Physiological microglia were divided into two clusters, while the microglia of ECM mice were divided into three clusters. Gene expression analysis showed that the M1 polarization gene of macrophages was higher in Cluster 7 of ECM mice than in Cluster 5 of normal mice (I), the monocyte genes increased in Cluster 9 compared with Cluster 7 of ECM mice (J), and most of the M1/M2 macrophage genes declined (K)

Transcriptome analysis of microglia in mice with ECM has been completed^3^; however, there are still some deficiencies in knowledge of the brain immune niches. To fill this gap, the brainstem regions of three mice with ECM in the infection group and two mice of the normal group were analyzed on days 7–9 after incubation using single‐cell RNA sequencing (RNA‐seq). RNA‐seq results showed that the expression of PD‐L1 increased significantly in the brainstem of mice with ECM compared with that in the normal group (Figure 4A), and several types of nerve cells expressed PD‐L1 (Figure 4B), among which microglia was one of the major cells expressing PD‐L1 in the brainstem (Figure 4B). In addition, astrocytes, neurons, and endothelial cells also expressed PD‐L1 in the ECM group (Figure 4B). The peripherally infiltrating CD8^+^ T cells were the main cells in the brainstem of ECM mice, revealing the upper level of PD‐1, and notably, microglia also expressed an amount of PD‐1 (Figure 4B). Further analysis revealed that the Ki67 of microglia in the brainstem of ECM mice was enhanced compared with that in normal mice, and the expression of CCL2 in the microglia also increased significantly (Figure 4C). It should be a concern that the proportion of CD45^high^ cells increased, while the proportion of CD45^int^ cells decreased (Figure 4D), showing that the expression of CD45 in microglia increased significantly after PbA infection. IF staining of brainstem tissue sections confirmed that CX3CR1‐GFP^+^ cells expressed PD‐L1 on the 7th day after infection (Figure 4E).

Further analysis of the microglia subgroups revealed that there was heterogeneity between the ECM mice and normal control mice (Figure 4F). Clustering analysis revealed four unique microglial subgroups across ECM pathological and physiological conditions. Cluster sizes ranged from 4.91% to 46.31% of total microglia, and Cluster 3 was almost entirely composed of microglia in the ECM brain and showed obvious transcriptional characteristics that were different from those in physiological conditions (Figure 4G). Analysis of microglia in the physiological state of the brain revealed that there were only two clusters, with the larger group (Cluster 5) accounting for more than 80% (Figure 4H). PbA infection caused a redistribution of microglial states and the microglia were divided into three groups, among which cluster 7 accounted for nearly 50%. Gene expression analysis between the two most abundant clusters in the two groups (Clusters 5 and 7) showed that the canonical microglial genes (Tmem119, Siglec‐H, P2ry12, and Trem2^17^) were downregulated and expressed by most of the microglia in the ECM mice, and the monocyte/macrophage markers (Itgam and Cx3cr1) showed lower levels in ECM but Cxcl2 was highly expressed (Figure 4I). The transcripts (Ccl2, Tnf, and Il1b) of the M1 polarization gene in macrophages were certainly expressed at much higher levels in Cluster 7 of the microglia in ECM mice, whereas the M2 polarization gene of macrophage (Csf1r) showed lower levels. Both the macrophage genes and the microglia genes reduced in Cluster 9 compared with Cluster 7, but most of the monocyte genes increased in Cluster 9 (Figure 4J). The M1 and M2 macrophage genes almost declined and were expressed by most of the Cluster 9 cells, but only one (Ccl5) was highly expressed relative to Cluster 9 compared with Cluster 7 (Figure 4K).

### 3.5 Specific enhancement of the PD‐1/PD‐L1 pathway in the brain protects against ECM

**FIGURE 5:**
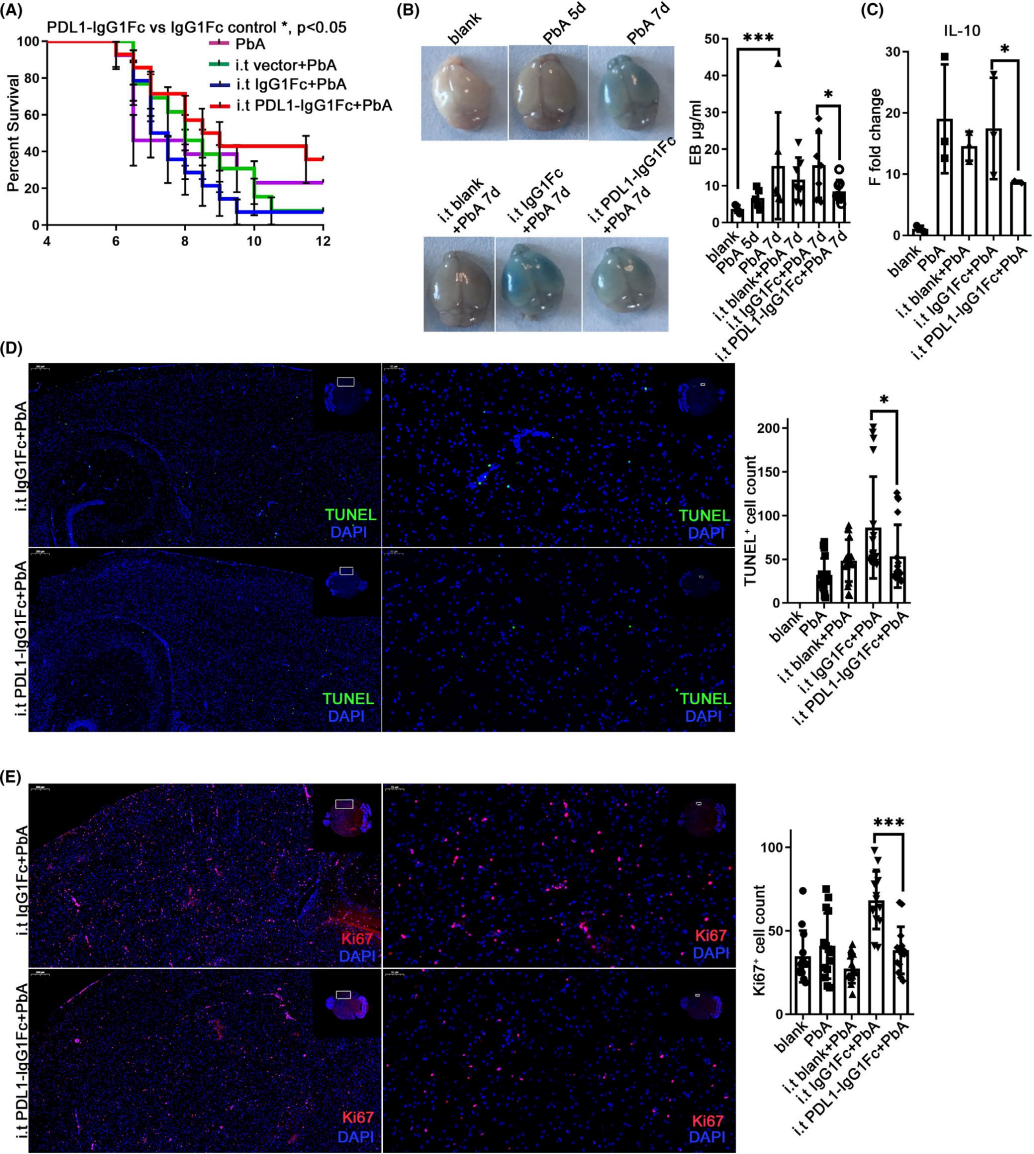
Enhancement of the PD‐1/PD‐L1 pathway in the brain protected against ECM. Intrathecal injection was performed at −1 dpi and 1 dpi. Survival was monitored daily. The survival time of the i.t PDL1‐IgG1Fc group is longer than that of the i.t IgG1Fc group (log‐rank test, *n* = 13, *p* = 0.0491) (A) and the improved opening of the BBB showed by EB injection at 7 dpi (B). (C) The mRNA level of IL‐10 decreased in the i.t PD‐L1 group compared with that in the IgG1Fc group. IF‐stained brain sections at 7 dpi indicate descended TUNEL (D) and Ki67 (E) staining. The results are expressed as the mean ± SD of three independent experiments. *p < 0.05, **p < 0.01 and ***p < 0.001 indicate that the differences are significant (unpaired *t*‐test, *n* = 3)

Intravenous injection of PDL1‐IgG1Fc fusion protein in mice can effectively inhibit the occurrence of ECM.^10^ However, systemic enhancement of the PD‐1/PD‐L1 signaling pathway may reduce the ability of the immune system to eliminate malaria parasites. To verify the protective effect of intracerebral‐specific PD‐1/PD‐L1 enhancement, an adenovirus expression vector was constructed to express the PDL1‐IgG1Fc fusion protein (Figure S4A) using adenovirus expressing IgG1Fc as controls. By intrathecal injection, viral vectors were distributed freely throughout the brain with cerebrospinal fluid (CSF),^18^ infected nerve cells spontaneously, and reached its peak expression in 3–4 days after infection. Adenovirus was intrathecally injected, and the level of PD‐L1 protein in the injected brain increased (*p* = 0.0006) compared with the non‐injected brain using western blotting (Figure S4B). Enhanced expression of PD‐L1 in the brain by intrathecal injection of adenovirus on day 1 and day 1 of PbA infection increased the survival rate of ECM mice (*p* = 0.0491) (Figure 5A). The EB exudation test was used to evaluate the integrity of the BBB, and the results showed that intrathecal injection of PDL1‐IgG1Fc significantly reduced the degree of BBB damage, and the amount of EB exudation decreased (*p* = 0.0128) compared with injection of IgG1Fc adenovirus (Figure 5B).

The effect of the intrathecal adenovirus expression vector on the PD‐1/PD‐L1 pathway was detected using qPCR. The transcription levels of IL‐10 in the brain of mice in the PDL1‐IgG1FC i.t. group showed a significant downregulation on the 7th day (*p* = 0.0364) after infection compared with that in the IgG1Fc adenovirus i.t group (Figure 5C), and there was no significant difference in the transcriptional level of inflammatory cytokine TNF‐α and IL‐6 (Figure S4C). IF staining showed the number of TUNEL‐positive cells in the ECM brain with PDL1‐IgG1Fc i.t. was lower than that with IgG1Fc i.t (*p* = 0.0498) (Figure 5D). However, the number of Ki67‐positive cells in the PDL1‐IgG1FC i.t. group decreased compared with that in the IgG1Fc i.t. group (*p *< 0.0001) (Figure 5E).

### 3.6 Intracerebral enhancement of PD‐L1 affected the immune microenvironment of each brain region

**FIGURE 6:**
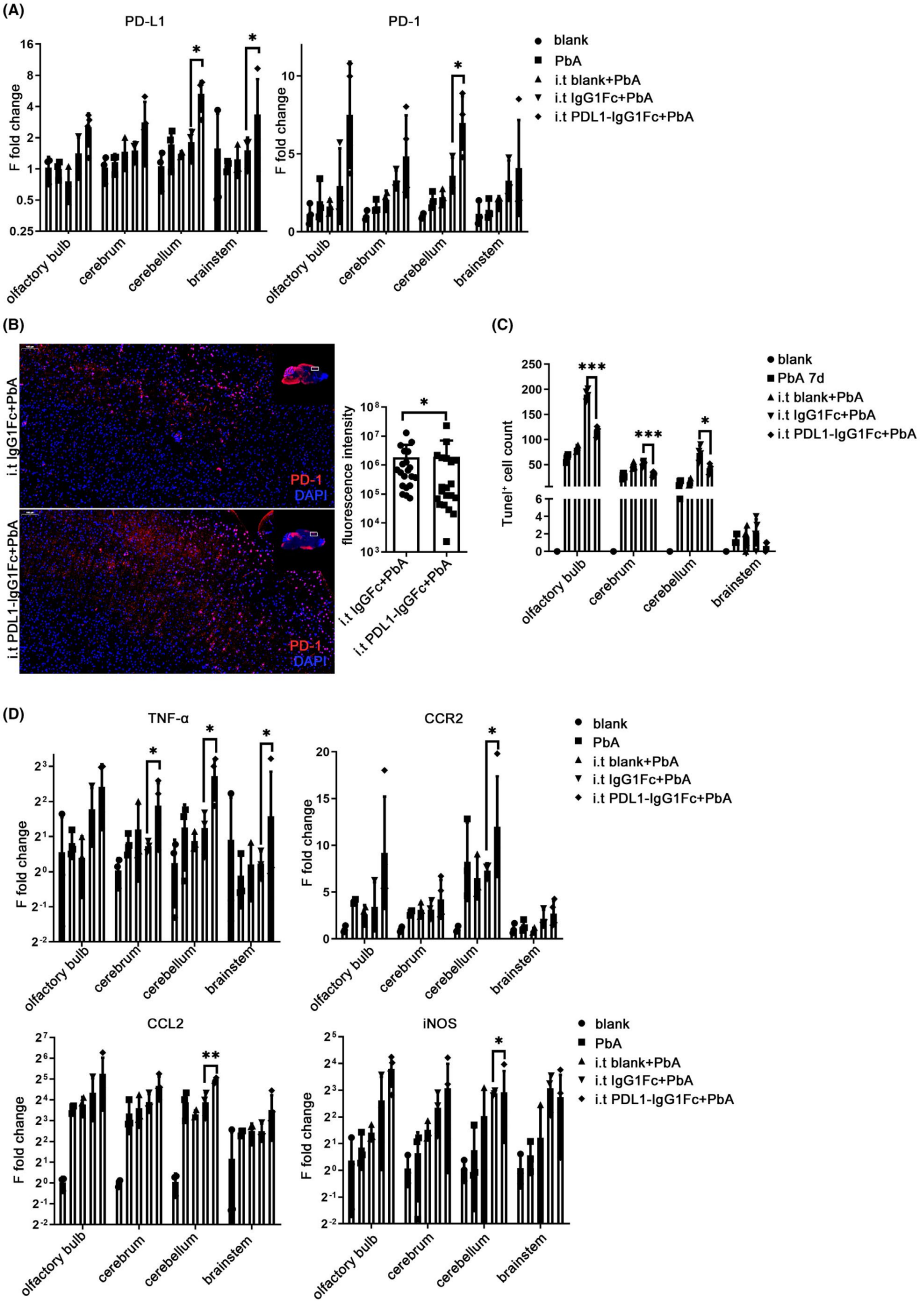
Intracerebral enhancement of PD‐L1 affected the immune microenvironment of each brain region. Intrathecal injection was performed at −1 dpi and 1 dpi on C57BL/6 mice. (A) The mRNA levels of PD‐L1 are upregulated in the cerebellum and brainstem while PD‐1 increased in the cerebellum of ECM mice injected with PDL1‐IgG1Fc, with the IgG1Fc group as control. IF‐stained brain sections at 7 dpi indicate increased PD‐1 (B) and decreased TUNEL (C) staining in the PDL1‐IgG1Fc group compared with that in the IgG1Fc group. (D) The changes in mRNA levels of inflammatory cytokines are shown in the brain of ECM mice injected with PDL1‐IgG1Fc compared with those in the IgG1Fc group. The results are expressed as the mean ± SD of three independent experiments. *p < 0.05, **p < 0.01, and ***p < 0.001 indicate that the differences are significant (unpaired *t*‐test, *n* = 3)

To further clarify the effect of the PD‐L1 fusion protein in different brain regions, qPCR was used to detect the transcriptional levels of PD‐1 and PD‐L1 in each brain region. The transcriptional level of PD‐L1 showed significant upregulation in the cerebellum (*p* = 0.0225) and brainstem (*p* = 0.0304) of ECM mice in the PDL1‐IgG1Fc i.t. group compared with that in the IgG1Fc i.t. group, while the significant change in PD‐1 was only found in the cerebellum (*p* = 0.0225) (Figure 6A). There was no difference in the protein levels of PD‐1 and PD‐L1 in the brainstem between the PDL1‐IgG1Fc group and the IgG1Fc group (Figure S5A).

IF staining of PD‐1 showed an increase in the PDL1‐IgG1Fc i.t. group compared with that in the IgG1Fc i.t. group (*p* = 0.0366) (Figure 6B). IF staining also confirmed that the number of TUNEL‐positive cells in the brain of ECM mice injected with PDL1‐IgG1Fc i.t. decreased significantly in the cerebrum (*p* < 0.0001), cerebellum (*p* = 0.0145), and olfactory bulb (*p* < 0.0001) compared with that in the IgG1Fc i.t. group (Figure 6C; Figure S5B). Analysis of the level of β‐APP and MCP‐1 in the brainstem using western blotting showed that there was no significant difference between the PDL1‐IgG1Fc i.t. group and IgG1Fc group (Figure S5C).

The results of qPCR indicated that the transcriptional level of TNF‐α increased in the cerebrum (*p* = 0.0160), cerebellum (*p* = 0.0417), and brainstem (*p* = 0.0117) in the PDL1‐IgG1Fc i.t. group compared with that in the IgG1Fc i.t. group, and the chemokines, including CCR2 (*p* = 0.0432), CCL2 (*p* = 0.0040), and iNOS (*p* = 0.0145), were upregulated in the cerebellum of the PDL1‐IgG1Fc i.t. group compared with those in the IgG1Fc i.t. group (Figure 6D). IF staining showed a higher level of IL‐6 in the brain of the PDL1‐IgG1Fc i.t. group than that in the IgG1Fc control group (*p* = 0.0002) (Figure S5D).

### 3.7 Intracerebral enhancement of PD‐L1 affects the activation and inflammation of microglia

**FIGURE 7:**
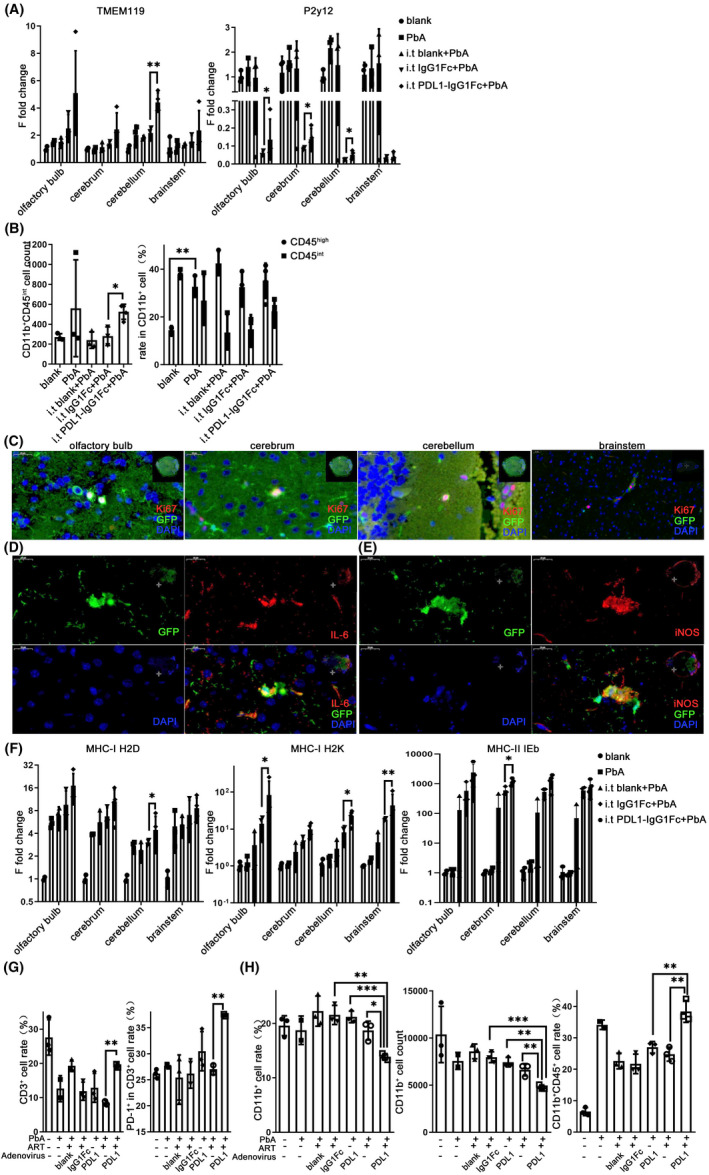
Intracerebral enhancement of PD‐L1 affected the activation and inflammation of microglia. C57BL/6 were injected intrathecally with recombinant adenovirus at −1 dpi and 1 dpi. (A) qPCR shows that the expression levels of TMEM119 increased in the cerebellum and P2Y12 increased in the olfactory bulb, cerebrum, and cerebellum of the PDL1‐IgG1Fc group, compared with those in the IgG1Fc group. (B) The flow cytometry was used to analyze the percentage of CD11b^+^ cells and the population of CD45^int^ cells in CD11b^+^ cells in the brain of the PDL1‐IgG1Fc i.t. group. (C–E) IF‐stained CX3CR1‐GFP brain sections at 7 dpi shows that the microglia expressed Ki67, IL‐6, and iNOS in the ECM mice with PDL1‐IgG1FC i.t. (F) qPCR shows that the expression levels of MHC‐I and MHC‐II increased in the PDL1‐IgG1Fc group. Combined use of artemisinin and intrathecal injection of recombinant adenovirus. Flow cytometry analysis shows that the proportion of PD‐1^+^ cells in CD3^+^ cells (G) and the proportion of CD45^+^ cells in CD11b^+^ cells (H) increased in the brain of mice treated with artemisinin and PDL1‐IgG1Fc i.t. compared with those in the artemisinin or PDL1‐IgG1Fc i.t. group. The results in (A–B and F–H) are expressed as the mean ± SD of three independent experiments. *p < 0.05, **p < 0.01, and ***p < 0.001 indicate that differences are significant (unpaired *t*‐test, *n* = 3)

On the 7th day after infection, the transcriptional levels of microglia‐specific markers were detected using qPCR. Compared with the IgG1Fc i.t. control group, the transcriptional level of TMEM119 increased significantly in the cerebellum of the PDL1‐IgG1Fc i.t. group (*p* = 0.0051), and P2Y12 increased markedly in the olfactory bulb (*p* = 0.0325), cerebrum (*p* = 0.0288), and cerebellum (*p* = 0.0183) of the PDL1‐IgG1Fc i.t. group (Figure 7A). Unexpectedly, P2Y12 also decreased in the IgG1Fc i.t. group, which may be related to the widespread distribution of Fc receptors on the surface of the microglia. There was no significant difference in the transcriptional level of Siglec‐H (Figure S6A). Both the percentage and quantity of CD11b^+^ cells in the ECM brain with PDL1‐IgG1Fc i.t. showed no significant difference on the 5th day (Figure S6B). The quantity of CD11b^+^CD45^int^ increased observably in the PDL1‐IgG1Fc i.t. group compared with that in the IgG1Fc i.t. group (*p* = 0.0118), while the proportion of CD45^int^ cells in CD11b^+^ cells showed no difference between the PDL1‐IgG1Fc i.t. and IgG1Fc i.t. groups (Figure 7B; Figure S6B).

IF staining of mouse brain sections confirmed that Ki67 expression was widely observed in the microglia of ECM mice in the PDL1‐IgG1Fc i.t. group, while the expression decreased in the IgG1Fc i.t. group (Figures 5E and 7C). CX3CR1‐GFP^+^ cells also expressed the inflammatory molecules IL‐6 and iNOS in the ECM brain with PDL1‐IgG1Fc i.t. (Figure 7D,7E). Compared to the ECM brain with IgG1Fc i.t., the transcriptional levels of MHC‐I H2D in ECM group with PDL1‐IgG1Fc i.t. increased significantly on the 7th day in the cerebellum (*p* = 0.0224); the MHC‐I H2K upregulated visibly in the olfactory bulb (*p* = 0.0102), cerebellum (*p* = 0.0470) and brainstem (*p* = 0.0015) (Figure 7F); the MHC‐II IEb showed a marked raise in the cerebrum (*p* = 0.0334) (Figure 7F).

Artemisinin as the priority drug for falciparum malaria is often unable to help patients with CM recover. To verify the adjuvant effect of PD‐L1 on artemisinin, the inflammatory effects of combined use were examined on ECM mice. The percentage of CD3^+^ T cells in the cells extracted from the brain showed a visible upregulation in the group treated with artemisinin and PDL1‐IgG1Fc i.t. compared with the group treated with artemisinin only (*p* = 0.0040), and the proportion of PD‐1^+^ cells in CD3^+^ cells also increased significantly in the combined treatment group relative to the artemisinin group (*p* = 0.0056) (Figure 7G; Figure S6C). The proportion of CD11b^+^ cells showed a significant decrease in the combined treatment group compared with the artemisinin group (*p* = 0.0104) or PD‐L1 (*p* = 0.0005) alone (Figure 7H; Figure S6D). Similarly, the quantity of CD11b^+^ cells decreased observably in the combined treatment group relative to the artemisinin group (*p* = 0.0097) or the PD‐L1 group (*p* = 0.0012) (Figure 7H; Figure S6D). Further analysis showed that the proportion of CD45^+^ in CD11b^+^ cells was upregulated in the combined treatment group compared with the artemisinin group (*p* = 0.0048) or the PD‐L1 group (*p* = 0.0074) (Figure 7H; Figure S6E) and the quantity of CD11b^+^CD45^+^ cells showed no difference (Figure S6F).

## 4 DISCUSSION

CM is one of the most serious complications caused by *Plasmodium falciparum* infection, with a mortality rate of 25%.^19^ Owing to the species specificity of *Plasmodium*, ECM in mice is a widely used alternative model of CM to study the pathogenesis. The death of ECM mice was related to cerebral vascular rupture, edema, and neuronal apoptosis. The plasmodium‐specific CD8^+^ T cells played an important role in the pathological process of ECM,^20^ such as migration to the brain^21^ and destruction of the endothelial cells.^22^ In this study, we observed CD8^+^ T cells and peripheral macrophages were the main infiltrating cells in the immune microenvironment of the brainstem. Although the macrophages were involved in the clearance of pRBCs in cerebral vessels, they did not play an important role in the development of cerebral malaria as CD8+ T cells.^20^ Therefore, immunomodulation of infiltrating CD8^+^ CTL cells has become a potential therapeutic direction for the prevention and treatment of CM.

The CNS is a relatively closed environment; however, studies have shown that resident cells and their microenvironment do actively modulate immune response after infection. This study confirmed the immune checkpoint molecules PD‐1 and PD‐L1 increased in ECM brain. Our single‐cell analysis confirmed that CD8^+^ T cells highly expressed PD‐1 in the brainstem of ECM mice. Our previous study has confirmed that *Pdcd1* ^−/−^ mice showed more severe ECM symptoms, while peripheral blood PDL1‐IgG1Fc interference inhibited the activation and cytotoxicity of CD8^+^ T cells,^20^ suggested that the interaction of PD‐1 and PD‐L1 leads to the activation of the immune tolerance pathway of T cells, which makes it an excellent target for CTL cell immunotherapy. However, the immune microenvironment needs to maintain a delicate balance between pro‐ and anti‐inflammatory immune response to control parasitemia properly without causing severe immunopathology, which requires precise control of the spatial and temporal aspects of the immune microenvironment in the brain.

Our single‐cell analysis also confirmed that the microglia were the main cells to express the PD‐L1 in the ECM brainstem. The use of microglia to regulate the immune microenvironment in the brain has become a new research direction. Studies on multiple sclerosis and other nervous system diseases have suggested that microglia played an important role in the pathogenesis of inflammation, demyelination, and nerve cell injury.^23^ In a nonlethal mouse malaria model, Iba1^+^ cells activated from the resting to the amoeba phase.^24^ This study showed that microglia activation‐related transcription factors increased continuously in various brain regions of ECM mice, the percentage and number of microglia increased with infection, and the proliferation signal and the expression of major chemokines in macrophages increased significantly. These results suggested that microglia could be activated and participated in the construction and maintenance of the immune niches.

However, the occurrence of many nervous system diseases was also related to the infiltration of peripheral monocytes. The monocyte‐derived macrophages could cross the BBB to infiltrate the brain; however, the expression profiles of myeloid macrophages and microglia were different.^17^, ^25^, ^26^ Our single‐cell analysis of the brainstem confirmed that both invading macrophages and microglia accounted for a large number of cells after infection, which implied that microglia may also have functional differences from the macrophages. CD45 was considered as a marker to differentiate microglia (CD11b^+^CD45^low^) from peripheral macrophages (CD11b^+^CD45^high^) using flow cytometry.^27^, ^28^ However, our flow cytometry and single‐cell sequencing results confirmed that CD45 expression in the microglia in ECM mice increased with infection, demonstrating that the activation of microglia was related to the increase in CD45 expression. Moreover, single‐cell analysis also found heterogeneous microglia subgroups, and it is worth further study whether subgroups have functional differences or even have special immune regulatory functions.^29^

More importantly, microglia, as the main cells expressing PD‐L1 in the brain immune microenvironment, were activated by the upregulation of PD‐L1 expression. However, the existing PD‐L1 level in the brain was difficult to inhibit the occurrence of ECM, so we tried to specifically enhance the PD‐L1 function in the brain to alleviate the inflammation. Owing to the lack of efficient and specific methods to regulate PD‐L1 expression in microglia, we enhanced the PD‐1/PD‐L1 signaling pathway through the intrathecal injection of recombinant adenovirus expressing PDL1‐IgG1Fc. Our results showed that increased expression of PD‐L1 in the brain significantly increased the survival rate of mice; reduced the incidence of ECM and the degree of BBB damage; downregulated the transcription levels of the inflammatory cytokines TNF‐α, IL‐6, and IL‐10; and decreased nerve cell death. PbA infection also induced microglia to upregulate the expression of PD‐1, suggesting that it could also be negatively regulated by immunity.

Aging and chronic inflammation could activate microglia; increase the pro‐inflammatory response, oxidative damage, and phagocytosis; and deliver antigens to infiltrating CD4^+^ T or CD8^+^ T cells through MHC‐I/II molecules.^30^ In response to effective communication with T cells, the phenotype of microglia could change from the active M1 type to the neuroprotective M2 type, which corresponded to tissue remodeling and homeostasis.^31^ The single‐cell RNA‐seq results suggested that microglia showed significant heterogeneity, and the expression of M1 polarized related genes of macrophages was upregulated. We found that intrathecal injection of PDL1‐IgG1Fc resulted in the reduction in microglia proliferation as well as the expression of the inflammatory molecules IL‐6 and iNOS; however, the expression of macrophage chemokines and macrophage activation increased. Unexpectedly, intrathecal injection of IgG1Fc also affected the activation of microglia, which may be related to the engagement of IgG1Fc and the Fc receptor of microglia. Combined PDL1‐IgG1Fc i.t. and artemisinin treatment inhibited the inflammatory function of T cells and reduced the number of microglia/macrophages in the brain, thus playing a protective role against ECM.

In conclusion, we confirmed that the maintenance of the immune microenvironment in the brain of mice with CM requires the participation of the negative immune regulatory pathway PD‐1/PD‐L1, and the activation and expression profile changes in microglia induced by ECM. Microglia, the main component of the brain immune microenvironment, were the main executive cells that regulated the neurotoxic effect of infiltrating CD8^+^ T cells through the PD‐1/PD‐L1 pathway. Intrathecal injection of PDL1‐IgG1Fc specifically upregulated the PD‐1/PDL1 signaling pathway in the brain, inhibited inflammation in the brain, reduced the death of nerve cells, and prevented the occurrence of ECM. The synergistic effect of PDL1‐IgG1Fc combined with artemisinin was also better than that of either alone. Our findings may provide a potential approach for an immune‐adjuvant therapy for human cerebral malaria.

## CONFLICTS OF INTEREST

The authors declare they have no conflicts of interest.

## AUTHORS’ CONTRIBUTIONS

YS, YZ, XAW, and YHL designed and supervised the study. YS, YHL, QHZ, JW, JL, and YXH performed the experiments and collected the data. YS, YHL, and QHZ summarized, analyzed, and plotted the data. YS wrote and finalized the manuscript. All authors have approved the final version of the manuscript.

## Supporting information

Fig S1

Fig S2

Fig S3

Fig S4

Fig S5

Fig S6

Table S1

Video S1

## Data Availability

The dataset generated and analyzed during the current study is available from the corresponding author upon reasonable request. Our original single‐cell RNA‐seq data have been submitted to the database of the NCBI Sequence Read Archive (http://www.ncbi.nlm.nih.gov/bioproj ect/) under the BioProject ID: PRJNA735877.
