# Supplementary figures and images for: The immunomodulatory effect of microglia on ECM neuroinflammation via the PD‐1/PD‐L1 pathway
Source: CNS Neurosci Ther. 2021 Nov 11;28(1):46–63. doi: 10.1111/cns.13760 (PMC8673706; doi:10.1111/cns.13760)

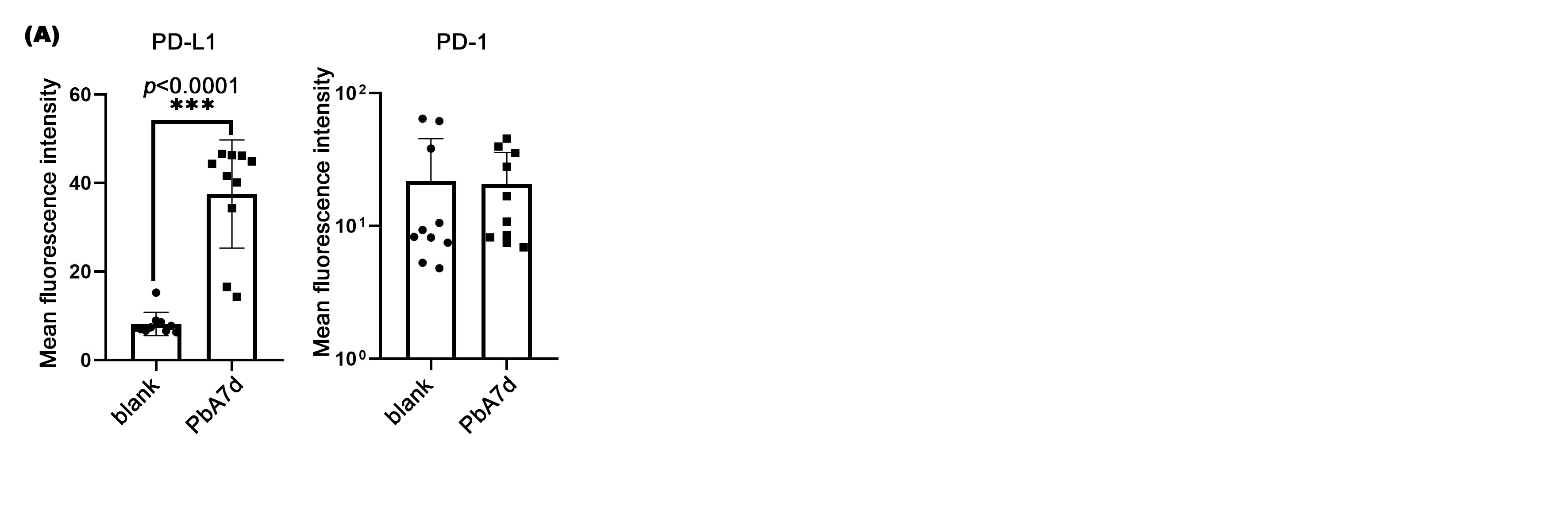

Supplement: Supplementary file 1 — Fig S1 [file CNS-28-46-s002.jpg]

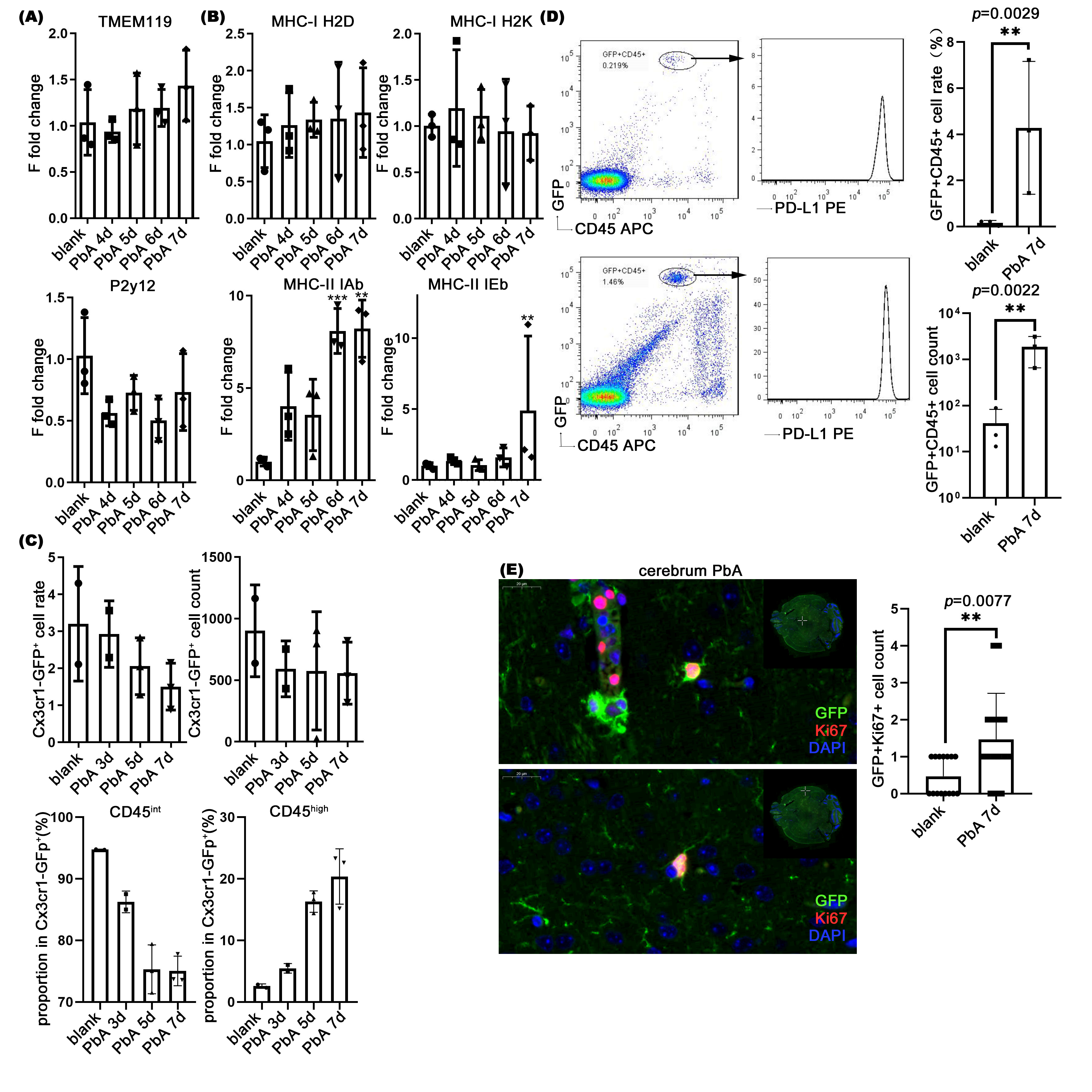

Supplement: Supplementary file 2 — Fig S2 [file CNS-28-46-s004.jpg]

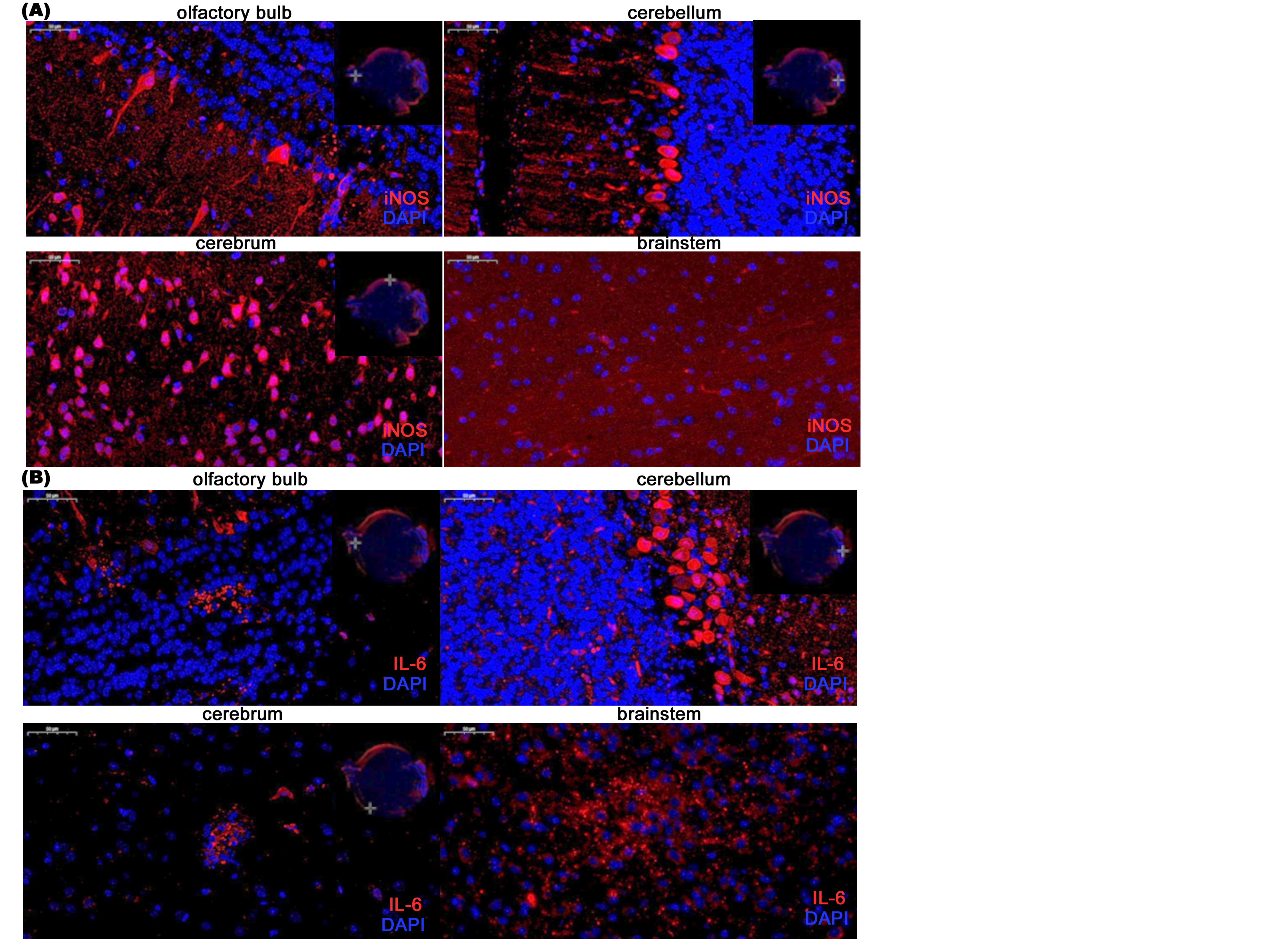

Supplement: Supplementary file 3 — Fig S3 [file CNS-28-46-s005.jpg]

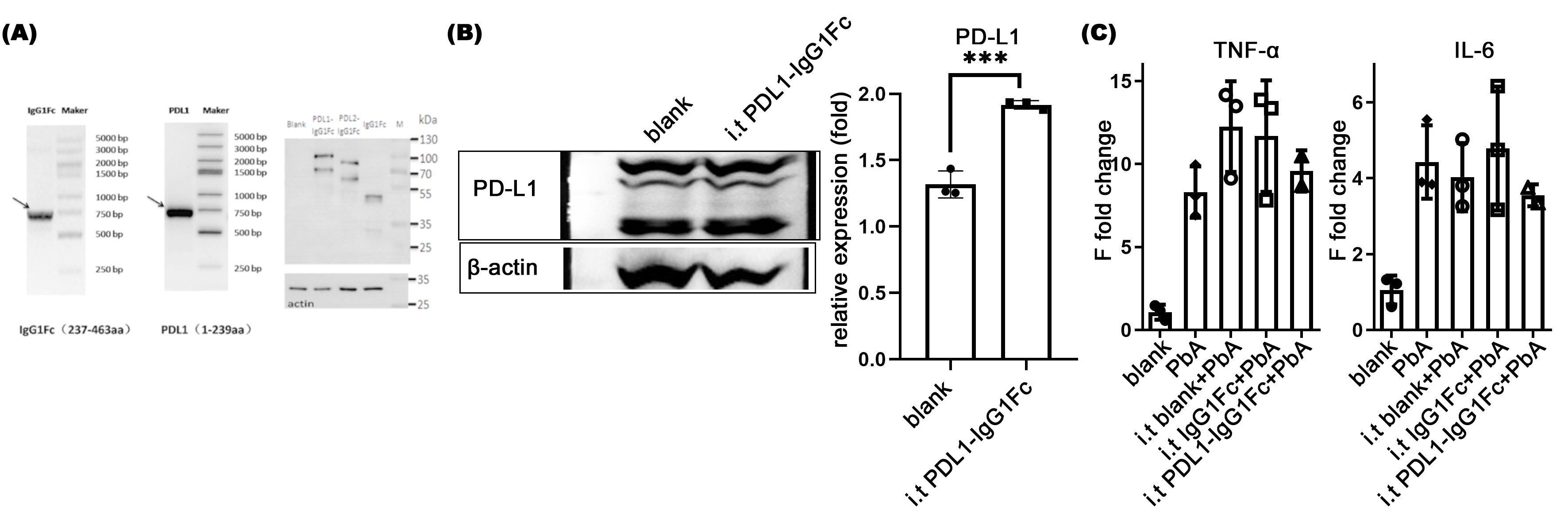

Supplement: Supplementary file 4 — Fig S4 [file CNS-28-46-s007.jpg]

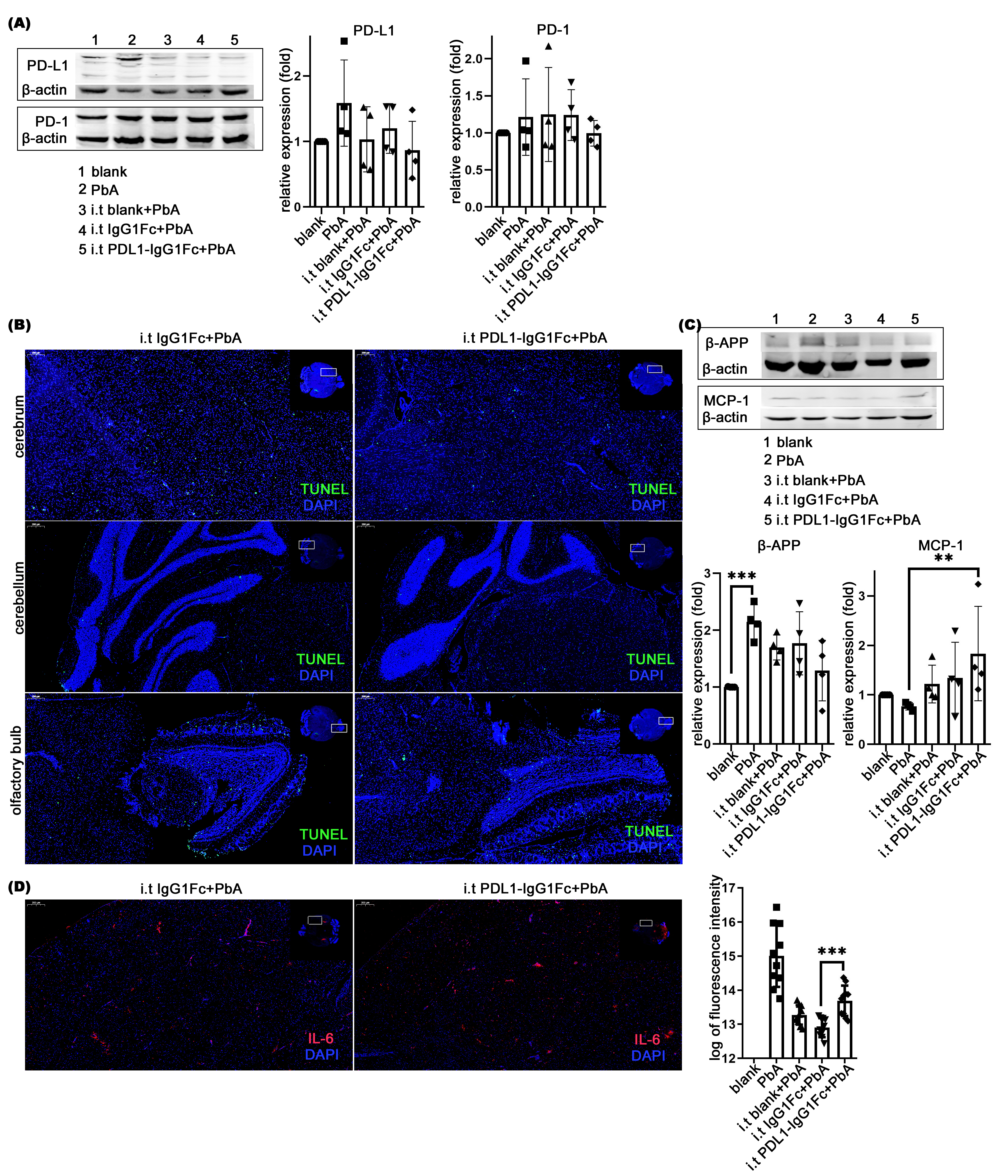

Supplement: Supplementary file 5 — Fig S5 [file CNS-28-46-s001.jpg]

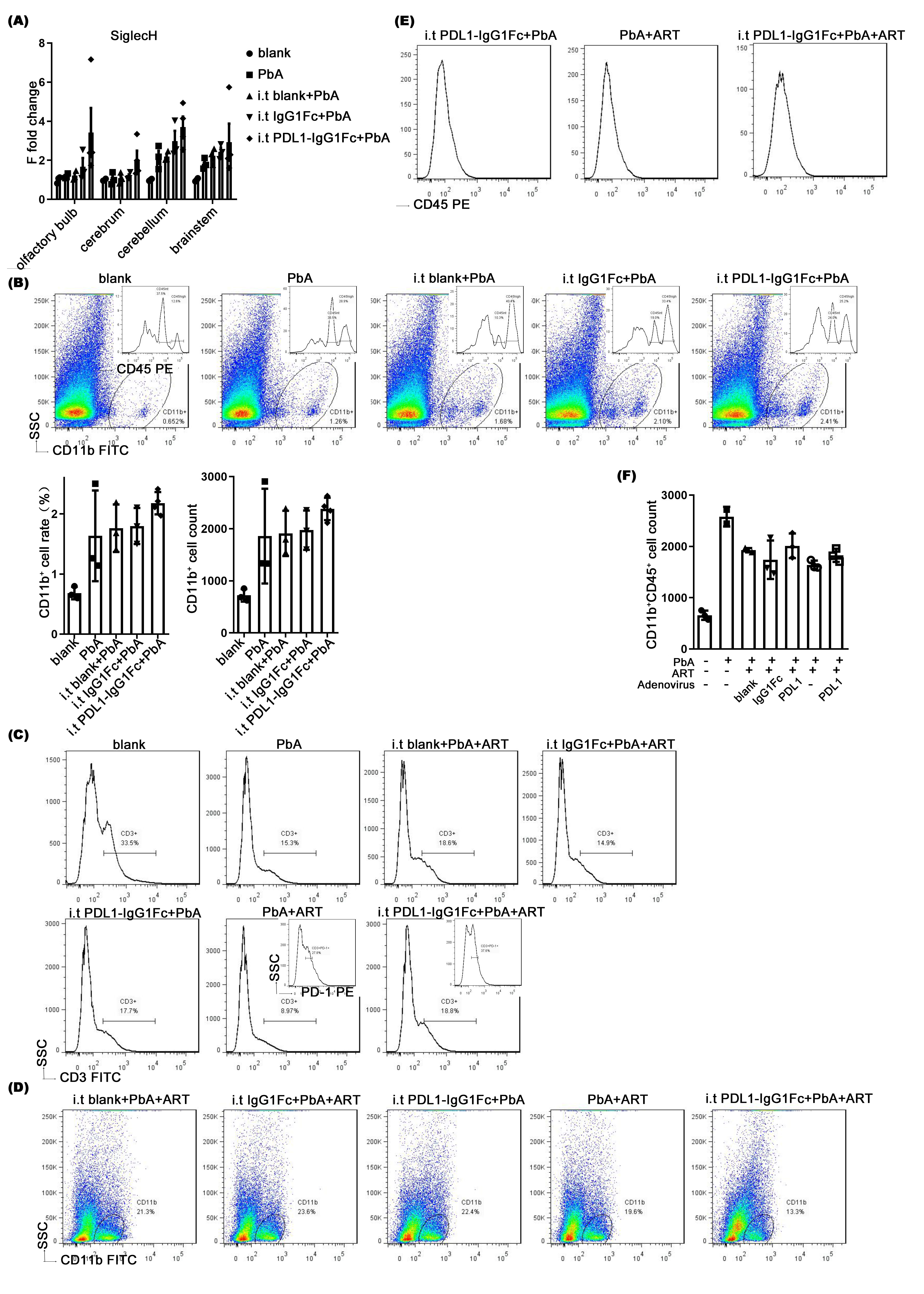

Supplement: Supplementary file 6 — Fig S6 [file CNS-28-46-s006.jpg]
