# Supplementary material for: The immunomodulatory effect of microglia on ECM neuroinflammation via the PD‐1/PD‐L1 pathway
Source: CNS Neurosci Ther. 2021 Nov 11;28(1):46–63. doi: 10.1111/cns.13760 (PMC8673706; doi:10.1111/cns.13760)
Supplement: Supplementary file 7 — Table S1 [file CNS-28-46-s008.docx]

Supplemental Table 1. The sequences of primers used for quantitative RT-PCR

| PD-1 | CAGCTTGTCCAACTGGTCG  GCTCAAACCATTACAGAAGGCG | PD-L1 | AGTATGGCAGCAACGTCACG  TCCTTTTCCCAGTACACCACTA |
| --- | --- | --- | --- |
| CCL2 | AGCAGCAGGTGTCCCAAAGA  GTGCTGAAGACCTTAGGGCAGA | CCR2 | AAGTTCAGCTGCCTGCAAAGAC  TCATCAAGCTCTTGGATACTTCGTG |
| Tmem119 | TGCATCTCGGCTGTGGAGAC  AGCGACATTGCAGGTGATGG | P2ry12 | TGCCAGTCTGCAAGTTCCACTAA  GGTGTTGACACCAGGCACATC |
| SiglecH | GAACTCCACAGCCCATGTCTCT  GCAGGAATTTGATAGCCGCTTCT | beta-actin | CATCCGTAAAGACCTCTATGCCAA C  ATGGAGCCACCGATCCACA |
| TNF-a | CAG GAG GGA GAA CAG AAA CTC CA  CCT GGT TGG CTG CTT GCT T | IL-10 | CCC TTT GCT ATG GTG TCC TT  TGG TTT CTC TTC CCA AGA CC |
| IL-6 | CCA CTT CAC AAG TCG GAG GCT TA  GCA AGT GCA TCA TCG TTG TTC ATA C | MHC-I H-2K | GATGCAGAGCATTACAGGGC  CGCTGGTAAGTGTGAGAGCC |
| MHC-I H-2D | GATGCAGAGCATTACAGGGC  GCCAGGTCAGGGCAATGTC | MHC-II IAb | ACAGCTTATTAGGAATGGGGACT  CACGGTGATGGGACTCTTCA |
| MHC-II IEb | AGCCTCTGGAACACCATAACC  CAGTCCGGTGGACACAACTC |  |  |
